# qPIPSA: Relating enzymatic kinetic parameters and interaction fields

**DOI:** 10.1186/1471-2105-8-373

**Published:** 2007-10-05

**Authors:** Razif R Gabdoulline, Matthias Stein, Rebecca C Wade

**Affiliations:** 1 Molecular and Cellular Modeling Group, EML Research gGmbH, Schloss Wolfsbrunnenweg 33, Heidelberg, 69118, Germany; 2 BIOMS (Center for Modeling and Simulation in the Biosciences), University of Heidelberg, Im Neuenheimer Feld 368, Heidelberg, 69120, Germany

## Abstract

**Background:**

The simulation of metabolic networks in quantitative systems biology requires the assignment of enzymatic kinetic parameters. Experimentally determined values are often not available and therefore computational methods to estimate these parameters are needed. It is possible to use the three-dimensional structure of an enzyme to perform simulations of a reaction and derive kinetic parameters. However, this is computationally demanding and requires detailed knowledge of the enzyme mechanism. We have therefore sought to develop a general, simple and computationally efficient procedure to relate protein structural information to enzymatic kinetic parameters that allows consistency between the kinetic and structural information to be checked and estimation of kinetic constants for structurally and mechanistically similar enzymes.

**Results:**

We describe qPIPSA: quantitative Protein Interaction Property Similarity Analysis. In this analysis, molecular interaction fields, for example, electrostatic potentials, are computed from the enzyme structures. Differences in molecular interaction fields between enzymes are then related to the ratios of their kinetic parameters. This procedure can be used to estimate unknown kinetic parameters when enzyme structural information is available and kinetic parameters have been measured for related enzymes or were obtained under different conditions. The detailed interaction of the enzyme with substrate or cofactors is not modeled and is assumed to be similar for all the proteins compared. The protein structure modeling protocol employed ensures that differences between models reflect genuine differences between the protein sequences, rather than random fluctuations in protein structure.

**Conclusion:**

Provided that the experimental conditions and the protein structural models refer to the same protein state or conformation, correlations between interaction fields and kinetic parameters can be established for sets of related enzymes. Outliers may arise due to variation in the importance of different contributions to the kinetic parameters, such as protein stability and conformational changes. The qPIPSA approach can assist in the validation as well as estimation of kinetic parameters, and provide insights into enzyme mechanism.

## Background

The ability to estimate enzymatic kinetic parameters is very important for metabolic simulations [1-3]. This is because experimental values of the kinetic parameters measured under exactly the conditions of the model for exactly the proteins in the model are usually missing. Often kinetic parameters have been measured only for a related enzyme or for the correct enzyme but under different conditions, e.g. different temperature, pH or ionic strength. Therefore we developed a method that relates variations in kinetic parameters to differences in protein structures. This method can be used to check the consistency of kinetic measurements described in the literature with available protein structural data as well as to make estimates of kinetic parameters based on enzyme structures and kinetic parameters for related enzymes.

Protein structural information provides a basis for predicting and rationalizing protein function. Given a protein structure, molecular simulations can be performed and these can be used to compute kinetic parameters [4]. For example, Brownian dynamics simulations can be used to simulate substrate-enzyme diffusional association and compute bimolecular association rate constants [5,6]. We previously demonstrated how rate constants computed by Brownian dynamics simulation of the diffusional association of superoxide and myeloperoxidase could be used in the mathematical modeling and simulation of the oscillatory behaviour of metabolite levels in activated white blood cells [2]. The type of molecular simulation to use must be chosen according to the mechanism determining the kinetic parameter. Whereas Brownian dynamics is appropriate for diffusional processes, molecular dynamics techniques may be required to simulate conformational changes and quantum mechanics for chemical reaction steps. These simulations can be computationally demanding and the accurate computation of kinetic parameters by simulations is a challenging and on-going research topic [4]. Therefore, a simpler, less computationally demanding and more robust approach to exploit protein structural information is required in the context of biochemical network simulation. qPIPSA is designed to fulfill this requirement.

In qPIPSA, molecular descriptors are related to kinetic parameters. The molecular descriptors are the molecular interaction fields (MIFs) of the proteins. Molecular interaction fields map the interaction energy between a chemical probe and the target protein as the chemical probe is moved over a grid of points [7]. Diverse chemical probes may be used, e.g. a water molecule, carbonyl oxygen or hydroxyl group. When the probe is a point charge, the molecular interaction field corresponds to the molecular electrostatic potential. Intermolecular interactions are fundamental to enzymatic reactions and are dependent on the enzyme MIFs. MIFs are often used in 3D-QSAR studies to derive quantitative structure-activity relationships (QSARs) [7]. This approach may be taken for proteins [8] as well as for small molecules [9,10]. The QSARs are generally derived by partial least squares (PLS) chemometric procedures for a training set with experimentally determined parameters. While such training procedures can be applied to predict enzyme parameters [11], typically, insufficient experimental data on kinetic parameters are available for training by PLS. In qPIPSA, we therefore employ a simpler linear regression procedure for which only two experimental measurements of a kinetic parameter for enzymes and at least one known three-dimensional structure are required. Further experimental measurements can be used and will help to improve the accuracy of predictions and assess the confidence of the parameter estimates. qPIPSA is based on the PIPSA method [12] which has been used to classify the interaction properties of protein families using MIFs [13,14].

In this paper, we will focus on the use of molecular electrostatic potential as the descriptor MIF in qPIPSA. The molecular electrostatic potential is usually the most informative MIF for this purpose. The long-range nature of electrostatic interactions means that similarities or differences in electrostatic potentials are often not detected in a sequence analysis. Even proteins with low sequence similarity can have rather similar electrostatic potentials [12,15]. For estimating enzyme kinetic parameters, the molecular electrostatic potential is appropriate because electrostatics are considered to be the most important contributor to enzymes' catalytic abilities [16], e.g. to stabilization of the transition state. Electrostatic steering has also been shown to enhance the association rates in fast, diffusion-influenced enzymes, such as superoxide dismutase [17].

In qPIPSA, the MIFs of different proteins and/or the same protein under different conditions, e.g. in a different titration state at a different pH, are compared. The enzyme kinetic parameters k_cat _and K_m _are associated with specific substrate-enzyme interactions. For the binding process relevant to the kinetic parameter, only one binding partner (here the enzyme) is modeled. The ligand is thus assumed to be the same or similar for all the protein structures compared. The differences in kinetic parameters are assumed to be determined by differences in the protein MIFs, and these differences are calculated in a region around the active site of the enzyme. Thus, qPIPSA requires no prior knowledge of the reaction mechanism. However, information about the location of the active site or substrate or ligand binding residues is used to define the region over which to compare MIFs. qPIPSA can be used to estimate missing kinetic parameters, to check the consistency of different measurements, and to investigate the mechanistic determinants of kinetic parameters. As such, it is a useful tool for biochemistry studies in general and for biochemical network simulation and comparative systems biology in particular.

In this paper, we first outline the qPIPSA approach. Details are given in the Methods section. Results are described for four illustrative and experimentally well-characterized enzymes. The criteria for choosing these enzymes were the availability of enough experimental kinetic and structural data for validation of the methodology, and a reasonable diversity in enzyme type. First, we present an analysis of acetylcholinesterase (AChE) mutants. This shows that not only inhibitor association rates but also substrate K_m _and k_cat_/K_m _parameters correlate remarkably well with the electrostatic potential differences near the active site of the enzyme. We then analyze superoxide dismutase (SOD) and show that the ionic strength and pH dependence of the rate constants for superoxide can be explained by the changes in the electrostatic potential. Next, we examine triose phosphate isomerase (TPI) enzymes from 12 different species. For TPIs, the electrostatic potential differences are found to be good descriptors for the cross-species variation in kinetic parameters k_cat_/K_m _and K_m_. In the fourth case, for 10 class I fructose-1,6-bisphosphate aldolases (FBA) the correlations are less obvious, but detectable. It appears that the conformation of the C-terminal region of FBA is critical for a description of the kinetics of this enzyme. Finally, we examine different protein structural modeling procedures and how to choose the comparison region for interaction fields, showing that this relates to enzyme mechanism.

### Theory

We postulate that the ratio of the kinetic parameters *k*_*a *_and *k*_*b *_of a pair of enzymes, *a *and *b*, correlates with the average differences in their molecular interaction fields (MIFs), Φ_*a *_and Φ_*b*_:

ln⁡(ka/kb)~α⋅∑R(Φa−Φb)/∑R1
 MathType@MTEF@5@5@+=feaafiart1ev1aaatCvAUfKttLearuWrP9MDH5MBPbIqV92AaeXatLxBI9gBaebbnrfifHhDYfgasaacH8akY=wiFfYdH8Gipec8Eeeu0xXdbba9frFj0=OqFfea0dXdd9vqai=hGuQ8kuc9pgc9s8qqaq=dirpe0xb9q8qiLsFr0=vr0=vr0dc8meaabaqaciaacaGaaeqabaqabeGadaaakeaacyGGSbaBcqGGUbGBcqGGOaakcqWGRbWAdaWgaaWcbaGaemyyaegabeaakiabc+caViabdUgaRnaaBaaaleaacqWGIbGyaeqaaOGaeiykaKIaeiOFa4hcciGae8xSdeMaeyyXIC9aaabuaeaacqGGOaakcqqHMoGrdaWgaaWcbaGaemyyaegabeaakiabgkHiTiabfA6agnaaBaaaleaacqWGIbGyaeqaaOGaeiykaKcaleaacqWHsbGuaeqaniabggHiLdGccqGGVaWldaaeqbqaaiabigdaXaWcbaGaeCOuaifabeqdcqGHris5aaaa@4E5F@

where **R **is the region selected for comparison and considered important for the kinetic parameter *k*. The differences in MIF, Φ, are summed over the grid points in the "skins" (see Methods section for a definition) around the proteins within a distance R from a specified point, defining the region **R **and divided by the number of points in the overlapping skins to obtain a size-independent measure.

When applied to a set of proteins, the correlation (1) is required for all pairs with known kinetic parameters, and the correlation factor *α *is derived by minimizing the function

∑ab{ln⁡(ka/kb)−α⋅∑R(Φa−Φb)/∑R1}2
 MathType@MTEF@5@5@+=feaafiart1ev1aaatCvAUfKttLearuWrP9MDH5MBPbIqV92AaeXatLxBI9gBaebbnrfifHhDYfgasaacH8akY=wiFfYdH8Gipec8Eeeu0xXdbba9frFj0=OqFfea0dXdd9vqai=hGuQ8kuc9pgc9s8qqaq=dirpe0xb9q8qiLsFr0=vr0=vr0dc8meaabaqaciaacaGaaeqabaqabeGadaaakeaadaaeqbqaaiabcUha7jGbcYgaSjabc6gaUjabcIcaOiabdUgaRnaaBaaaleaacqWGHbqyaeqaaOGaei4la8Iaem4AaS2aaSbaaSqaaiabdkgaIbqabaGccqGGPaqkcqGHsisliiGacqWFXoqycqGHflY1daaeqbqaaiabcIcaOiabfA6agnaaBaaaleaacqWGHbqyaeqaaOGaeyOeI0IaeuOPdy0aaSbaaSqaaiabdkgaIbqabaGccqGGPaqkaSqaaiabdkfasbqab0GaeyyeIuoakiabc+caVmaaqafabaGaeGymaedaleaacqWGsbGuaeqaniabggHiLdGccqGG9bqFdaahaaWcbeqaaiabikdaYaaaaeaacqWGHbqycqWGIbGyaeqaniabggHiLdaaaa@5699@

The relative percentage correlation error is defined using the RMSD of the left-hand side of (1) from the right-hand side and it is given by formula (3), with *α *minimizing (2):

100%⋅∑ab{ln⁡(ka/kb)−α⋅∑R(Φa−Φb)/∑R1}2/∑ab(ln⁡(ka/kb))2
 MathType@MTEF@5@5@+=feaafiart1ev1aaatCvAUfKttLearuWrP9MDH5MBPbIqV92AaeXatLxBI9gBaebbnrfifHhDYfgasaacH8akY=wiFfYdH8Gipec8Eeeu0xXdbba9frFj0=OqFfea0dXdd9vqai=hGuQ8kuc9pgc9s8qqaq=dirpe0xb9q8qiLsFr0=vr0=vr0dc8meaabaqaciaacaGaaeqabaqabeGadaaakeaacqaIXaqmcqaIWaamcqaIWaamcqGGLaqjcqGHflY1daGcaaqaamaaqafabaGaei4EaSNagiiBaWMaeiOBa4MaeiikaGIaem4AaS2aaSbaaSqaaiabdggaHbqabaGccqGGVaWlcqWGRbWAdaWgaaWcbaGaemOyaigabeaakiabcMcaPiabgkHiTGGaciab=f7aHjabgwSixpaaqafabaGaeiikaGIaeuOPdy0aaSbaaSqaaiabdggaHbqabaGccqGHsislcqqHMoGrdaWgaaWcbaGaemOyaigabeaakiabcMcaPaWcbaGaemOuaifabeqdcqGHris5aOGaei4la8YaaabuaeaacqaIXaqmaSqaaiabdkfasbqab0GaeyyeIuoakiabc2ha9naaCaaaleqabaGaeGOmaidaaOGaei4la8YaaabuaeaacqGGOaakcyGGSbaBcqGGUbGBcqGGOaakcqWGRbWAdaWgaaWcbaGaemyyaegabeaakiabc+caViabdUgaRnaaBaaaleaacqWGIbGyaeqaaOGaeiykaKIaeiykaKYaaWbaaSqabeaacqaIYaGmaaaabaGaemyyaeMaemOyaigabeqdcqGHris5aaWcbaGaemyyaeMaemOyaigabeqdcqGHris5aaWcbeaaaaa@7038@

The absolute error may be defined as:

∑ab{ln⁡(ka/kb)−α⋅∑R(Φa−Φb)/∑R1}2/∑ab1
 MathType@MTEF@5@5@+=feaafiart1ev1aaatCvAUfKttLearuWrP9MDH5MBPbIqV92AaeXatLxBI9gBaebbnrfifHhDYfgasaacH8akY=wiFfYdH8Gipec8Eeeu0xXdbba9frFj0=OqFfea0dXdd9vqai=hGuQ8kuc9pgc9s8qqaq=dirpe0xb9q8qiLsFr0=vr0=vr0dc8meaabaqaciaacaGaaeqabaqabeGadaaakeaadaGcaaqaamaaqafabaGaei4EaSNagiiBaWMaeiOBa4MaeiikaGIaem4AaS2aaSbaaSqaaiabdggaHbqabaGccqGGVaWlcqWGRbWAdaWgaaWcbaGaemOyaigabeaakiabcMcaPiabgkHiTGGaciab=f7aHjabgwSixpaaqafabaGaeiikaGIaeuOPdy0aaSbaaSqaaiabdggaHbqabaGccqGHsislcqqHMoGrdaWgaaWcbaGaemOyaigabeaakiabcMcaPaWcbaGaemOuaifabeqdcqGHris5aOGaei4la8YaaabuaeaacqaIXaqmaSqaaiabdkfasbqab0GaeyyeIuoakiabc2ha9naaCaaaleqabaGaeGOmaidaaOGaei4la8YaaabuaeaacqaIXaqmaSqaaiabdggaHjabdkgaIbqab0GaeyyeIuoaaSqaaiabdggaHjabdkgaIbqab0GaeyyeIuoaaSqabaaaaa@5D5A@

It gives an average deviation of the fit in ln units, but is less useful than expression (3) when comparing different cases with different degrees of kinetic parameter deviation. It is, for example, small in the absence of correlation when kinetic constants differ insignificantly in a set of proteins compared. We also used the Pearson correlation coefficient (R-coefficient) to estimate the degree of overall correlation between two sets of parameters x and y:

r=〈(x−〈x〉)(y−〈y〉)〉/〈(x−〈x〉)2〉〈(y−〈y〉)2〉
 MathType@MTEF@5@5@+=feaafiart1ev1aaatCvAUfKttLearuWrP9MDH5MBPbIqV92AaeXatLxBI9gBaebbnrfifHhDYfgasaacH8akY=wiFfYdH8Gipec8Eeeu0xXdbba9frFj0=OqFfea0dXdd9vqai=hGuQ8kuc9pgc9s8qqaq=dirpe0xb9q8qiLsFr0=vr0=vr0dc8meaabaqaciaacaGaaeqabaqabeGadaaakeaacqWGYbGCcqGH9aqpdaaadaqaaiabcIcaOiabdIha4jabgkHiTmaaamaabaGaemiEaGhacaGLPmIaayPkJaGaeiykaKIaeiikaGIaemyEaKNaeyOeI0YaaaWaaeaacqWG5bqEaiaawMYicaGLQmcacqGGPaqkaiaawMYicaGLQmcacqGGVaWldaGcaaqaamaaamaabaGaeiikaGIaemiEaGNaeyOeI0YaaaWaaeaacqWG4baEaiaawMYicaGLQmcacqGGPaqkdaahaaWcbeqaaiabikdaYaaaaOGaayzkJiaawQYiamaaamaabaGaeiikaGIaemyEaKNaeyOeI0YaaaWaaeaacqWG5bqEaiaawMYicaGLQmcacqGGPaqkdaahaaWcbeqaaiabikdaYaaaaOGaayzkJiaawQYiaaWcbeaaaaa@556E@

with <> being an average over all possible values of x or y.

Predictions of kinetic parameters are carried out by first deriving the parameter *α *for all pairs with known kinetic parameters and then averaging predictions for any unknown case from pairwise comparisons to all proteins with known kinetic parameters. The minimum required number of known parameters equals two in this approach.

When Φ is the molecular electrostatic potential, a physically meaningful estimate for the parameter *α *is Φ is -*q*/k_B_T, where *q *is the net charge of the substrate, k_B _is the Boltzmann constant and T is the temperature. This corresponds to the kinetic parameter being determined by the interaction energy of the substrate charge *q *with the electrostatic potential Φ of the enzyme: *k *~ *exp*(-*q*·Φ/k_B_T).

We also tested other possibilities for defining correlations, including

ln⁡(ka/kb)~α⋅ln⁡(∑Re±Φa/∑Re±Φb)
 MathType@MTEF@5@5@+=feaafiart1ev1aaatCvAUfKttLearuWrP9MDH5MBPbIqV92AaeXatLxBI9gBaebbnrfifHhDYfgasaacH8akY=wiFfYdH8Gipec8Eeeu0xXdbba9frFj0=OqFfea0dXdd9vqai=hGuQ8kuc9pgc9s8qqaq=dirpe0xb9q8qiLsFr0=vr0=vr0dc8meaabaqaciaacaGaaeqabaqabeGadaaakeaacyGGSbaBcqGGUbGBcqGGOaakcqWGRbWAdaWgaaWcbaGaemyyaegabeaakiabc+caViabdUgaRnaaBaaaleaacqWGIbGyaeqaaOGaeiykaKIaeiOFa4hcciGae8xSdeMaeyyXIC9aaSGbaeaacyGGSbaBcqGGUbGBcqGGOaakdaaeqbqaaiabdwgaLnaaCaaaleqabaGaeyySaeRaeuOPdy0aaSbaaWqaaiabdggaHbqabaaaaaWcbaGaeCOuaifabeqdcqGHris5aaGcbaWaaabuaeaacqWGLbqzdaahaaWcbeqaaiabgglaXkabfA6agnaaBaaameaacqWGIbGyaeqaaaaaaSqaaiabhkfasbqab0GaeyyeIuoaaaGccqGGPaqkaaa@554C@

For a single point in the region **R**, formulas (1) and (6) give the same results. When there are many points in the comparison region **R**, formula (6) will enhance the contributions from large and positive (+ sign) or large and negative (- sign) potentials. In some cases, formula (6) gives a better description of interaction field – kinetic parameter correlations.

Similarity indices, e.g. the Hodgkin index [18]

SIab=2⋅∑RΦaΦb/(∑RΦa2+∑RΦb2)
 MathType@MTEF@5@5@+=feaafiart1ev1aaatCvAUfKttLearuWrP9MDH5MBPbIqV92AaeXatLxBI9gBaebbnrfifHhDYfgasaacH8akY=wiFfYdH8Gipec8Eeeu0xXdbba9frFj0=OqFfea0dXdd9vqai=hGuQ8kuc9pgc9s8qqaq=dirpe0xb9q8qiLsFr0=vr0=vr0dc8meaabaqaciaacaGaaeqabaqabeGadaaakeaacqWGtbWucqWGjbqsdaWgaaWcbaGaemyyaeMaemOyaigabeaakiabg2da9iabikdaYiabgwSixpaaqafabaGaeuOPdy0aaSbaaSqaaiabdggaHbqabaaabaGaeCOuaifabeqdcqGHris5aOGaeuOPdy0aaSbaaSqaaiabdkgaIbqabaGccqGGVaWlcqGGOaakdaaeqbqaaiabfA6agnaaDaaaleaacqWGHbqyaeaacqaIYaGmaaGccqGHRaWkaSqaaiabhkfasbqab0GaeyyeIuoakmaaqafabaGaeuOPdy0aa0baaSqaaiabdkgaIbqaaiabikdaYaaaaeaacqWHsbGuaeqaniabggHiLdGccqGGPaqkaaa@5146@, may also be used to compare electrostatic potentials [13]. Similarity indices can assist in assigning a kinetic parameter to a protein when experimental values are available for several related proteins by enabling the most similar protein to be found and hence the kinetic parameter to be estimated [19]. For a more quantitative analysis, a pairwise distance matrix with 1−SIab
 MathType@MTEF@5@5@+=feaafiart1ev1aaatCvAUfKttLearuWrP9MDH5MBPbIqV92AaeXatLxBI9gBaebbnrfifHhDYfgasaacH8akY=wiFfYdH8Gipec8Eeeu0xXdbba9frFj0=OqFfea0dXdd9vqai=hGuQ8kuc9pgc9s8qqaq=dirpe0xb9q8qiLsFr0=vr0=vr0dc8meaabaqaciaacaGaaeqabaqabeGadaaakeaadaGcaaqaaiabdgdaXiabgkHiTiabdofatjabdMeajnaaBaaaleaacqWGHbqycqWGIbGyaeqaaaqabaaaaa@33A2@ as a distance measure can be constructed. We have previously shown how such a distance matrix can be used to compute relative electron transfer rates between plastocyanin and cytochrome f for a set of plastocyanin mutants based on the known electron transfer rate for wild-type plastocyanin [20]. The formulations based on similarity indices however suffer from loss of the sign information present in equation (1) and therefore do not allow a correlation to be made in a fully automated fashion. Equation (1) provides the most direct model in terms of the physical determinants of the quantities computed and has therefore been used in this manuscript. The correlations obtained with equation (1) are mostly of similar or better quality than those using similarity indices or distance matrices based on similarity indices.

The average Boltzmann factor of the ligand-protein interaction energy when the ligand is near or in the active site [21] is another possible descriptor of kinetic parameters. However, as the average Boltzmann factor is calculated from the interaction energy rather than the interaction field, it requires knowledge of the position and charge distribution of the ligand interacting with the enzyme. The use of MIFs alone in qPIPSA is less demanding in terms of modeling but can nevertheless be applied to comparing different enzymes.

## Results and discussions

### Molecular electrostatic potentials correlate with inhibitor association rate constants and substrate K_m _and k_cat_/K_m _rate constants for a set of acetylcholinesterase (AChE) mutants

**Figure 1:**
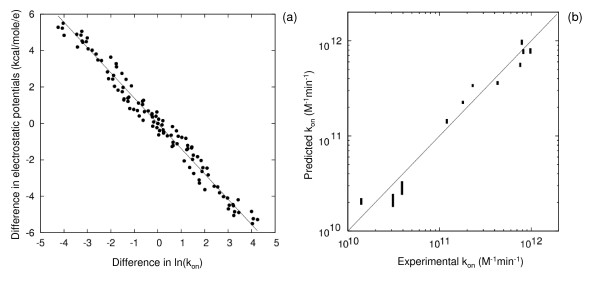
**(a) Correlation between the differences in experimental inhibitor ln(k_on_) and differences in electrostatic potentials of AchE and (b). leave-two-out cross-validation for prediction of k_on _values for the inhibitor TFK+ and AChE**. Each point in part (a) represents a pair of AChE variants for which the natural log (ln) of the difference in association rate constant, k_on_, for the inhibitor TFK+ is plotted on the x-axis and the average electrostatic potential difference in the comparison region is plotted on the y-axis. The straight line corresponds to the best linear fit and is given by y = -1.39*x. The data are for wild-type AChE and 10 mutants (see Table 1, no k_on_value for TFK+ is available for mutant 04). For leave-two-out cross-validation predictions presented in (b), 2 cases were omitted when deriving the correlation factor *α *in formula (1) and these 2 cases were predicted using formula (1) with the derived factor *α*. Predictions are shown as vertical lines connecting minimum and maximum values from all (55) different predictions.

We considered the wild-type mouse AChE and 11 mutants with large changes in kinetic parameters, see Table 1. The experimental kinetic data were taken from reference [22]. The electrostatic potential was computed for the 12 proteins and the average difference in electrostatic potential in the AChE active site gorge was then computed for all pairs of proteins. (See Methods section for a description of the exact region for MIF comparison). A remarkable correlation holds between the difference in ln(k_on_) (logarithm of association rate constant) of the inhibitor, m-trimethyl-ammonio-trifluoro-aceto-phenone (TFK+) with AChE, and the difference in the electrostatic potentials in the AChE active site gorge, see Fig. 1a. This correlation is consistent with the observations that on-rate constants correlate with the diffusional association rates calculated by Brownian dynamics simulations with electrostatic forces [23], and that diffusional association rates correlate with the average Boltzmann factor of the inhibitor-protein electrostatic interaction energy for positions of the inhibitor around the AChE active site [24]. Previously it was also shown that electrostatic potential values in different parts of the active site correlate with inhibitor k_on _[25].

**Table 1:** Kinetic data for mouse acetylcholinesterase (AChE) [22]

No	Mutation	k_on _TFK+ (10^11 ^M^-1^min^-1^)	k_cat_/K_m _ATCh (10^8 ^M^-1^min^-1^)	K_m _ATCh (μM)
00	WT	9.8 ± 0.6	30	46 ± 3
01*	D74N	0.39 ± 0.01	0.65	1300 ± 140
02*	E450Q	1.2 ± 0.1	0.24	140 ± 10
03*	E202Q	7.9 ± 0.4	4.3	200 ± 40
04*	D74N/E202Q/E450Q	-	0.0022	18000 ± 2500
05	D280V	8.2 ± 2.0	16	73 ± 4
06	D280V/D283N	7.6 ± 0.3	23	60 ± 9
07	E84Q/E91Q/D280V/D283N/D372N	2.3 ± 0.1	5.1	162 ± 6
08	E84Q/E91Q/D280V/D283N/E292Q/D372N	1.8 ± 0.1	2.3	230 ± 32
09	E84Q/E91Q/D280V/D283N	4.3 ± 0.8	4.6	240 ± 52
10*	D74N/E202Q	0.14 ± 0.01	0.34	700 ± 29
11*	D74N/D280V/D283N	0.31 ± 0.02 ^#^	0.23	1600 ± 320

The data are for wild-type and 11 mutant AChEs interacting with the inhibitor TFK+ and the substrate ATCh. Active site mutants are marked by an asterisk; mutant no. 11 has a combination of active site and surface residue mutations.

^#^The value published in reference [22] for this parameter is 10 times larger due to a typographical error [Z. Radic, personal communication].

As seen in Fig. 1a, a decrease in the average electrostatic potential by 1.39 kcal/mol/e results in an increase of k_on _for TFK+ by 1 natural log (ln) unit (equivalent to a factor of 2.72). This relation is in agreement with the expectation that ln(k_on_) is is a linear function of -Φ/k_B_T, where Φ is an average electrostatic potential in the comparison region. Standard LTO (leave-two-out) cross-validation to assess the predictive ability shows that accurate predictions of k_on _values can be obtained on the basis of this correlation, see Fig. 1b. Thus an excellent linear correlation between calculated differences in electrostatic potentials and measured ln(k_on_) values for TFK+ could be achieved and this can be used to predict ln(k_on_) values for AChE mutants.

**Figure 2:**
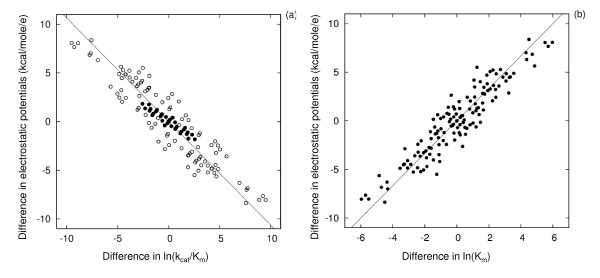
**Correlation between (a) experimental ln(k_cat _/K_m _and (b) experimental ln(K_m _for the substrate ATCh and electrostatic potential differences for different AChE mutants**. Each point corresponds to the differences for one protein variant pair. The straight line corresponds to the best linear fit and is given by y = -1.06*x (a) and y = 1.68*x (b). In the panel (a) data for protein pairs that do not have active site residue mutations are shown by filled circles.

A similar correlation was obtained for the difference in electrostatic potentials and the k_cat_/K_m _values of acetylthiocholine (ATCh), a substrate of AChE [22] (see Fig. 2a). Here, a decrease of the average electrostatic potential by 1.06 kcal/mol/e results in an increase of k_cat_/K_m _for acetylthiocholine by 1 ln unit. The overall correlation seems to be composed of different correlations. For surface residues (filled circles), changes in potential in the region of comparison result in larger changes in k_cat_/K_m _than for mutations of active site residues (open circles). This can be expected, because the surface residues influence k_cat_/K_m _not only via the potential in the active site comparison region but also because of the influence of the electrostatic potential of surface residues on the substrate as it approaches the active site.

The K_m _values for ATCh are also correlated with the AChE electrostatic potentials as shown in Fig. 2b. A decrease of the electrostatic potential by 1.68 kcal/mol/e results in a decrease of K_m _for ATCh by 1 ln unit.

Correlations for k_off _for TFK+ and k_cat _for ATCh (not shown) are significantly weaker. ln(k_cat_) is the sum of ln(k_cat_/K_m_) and ln(K_m_). Both of these quantities correlate with the electrostatic potential differences, but with opposite signs. Therefore, their sum appears to have a weak correlation with electrostatic potential differences.

**Figure 3:**
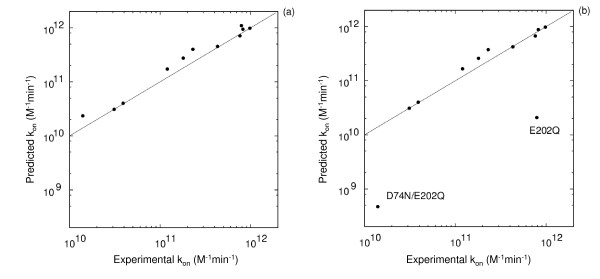
**Importance of correct modeling of the protonation states of titratable residues for predicting of k_on _values for TFK+ and AChE **. Predictions were made for 9 AChE mutants using known k_on _values for the wild-type and D74N/D280V/D283N mutant of AChE at pH 7. For the predictions on the left-hand side (a), residues E202 and H447 were modeled as singly and doubly protonated, respectively, while for the predictions on the right-hand side (b), E202 and H447 were modeled as unprotonated and singly protonated (at Nε), respectively.

An important finding here is that not only the inhibitor association rate constant but also the intrinsic enzyme kinetic parameters for a substrate can be correlated with the electrostatic potentials near the active site. The results are, however, sensitive to the modeling accuracy. We treated E202 and H447 as singly and doubly protonated, respectively (see Methods section). The importance of correct charge assignments for these residues was discussed previously [23] when performing Brownian dynamics simulations for this system. Assigning standard protonation states at pH 7 to these residues would weaken the correlation between electrostatic potential differences and inhibitor association rate constants and thus the predictive ability. For example, predictions made for 9 AChE mutants using known k_on _values for the wild-type and the D74N/D280V/D283N mutant of AChE and standard protonation states for E202 and H447 would result in a factor of 20 under-prediction of the inhibitor association rates for the mutants involving E202 (see Fig. 3b). The importance of charge assignments for these residues was discussed previously for Brownian dynamics simulations of this system. Only when the non-standard protonation states of these residues was assigned, was a good correlation between measured and calculated k_on _obtained (see Fig. 3a).

### The ionic strength and pH dependence of kinetic constants of Cu, Zn- superoxide dismutase (SOD) are reflected in molecular electrostatic potential

Kinetic constants measured under different environmental conditions can be correlated with MIFs computed for the respective conditions. Here, we demonstrate this for the rate constant for the first step of the SOD reaction which is limited by superoxide association to SOD. Bovine SOD was chosen because its reaction mechanism has been thoroughly studied [26] and the consistency of kinetic constants measured under different conditions has been analyzed [27]. The association rate of superoxide to SOD shows a pronounced pH- and ionic strength dependence. The pH was varied between 7.7 and 11.0 (at a constant ionic strength of 20 mM) and the ionic strength was varied between 20 and 250 mM at a constant pH of 7.7.

**Figure 4:**
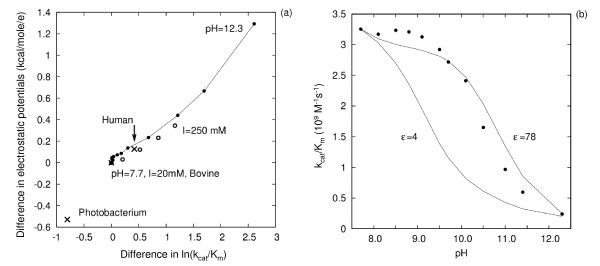
**(a) Correlation between experimental ln(k_cat _/K_m_) for superoxide and electrostatic potential differences for SOD from different organisms or under different conditions, and (b) experimental and calculated pH dependence of the rate constant for association of superoxide with bovine SOD**. (a) The reference point (0,0) marked by a cross is for bovine SOD at pH 7.7 and 20 mM ionic strength with corresponding experimental rate constant of 3.8 10^9^M^-1^s^-1 ^[26]. The other 2 crosses are for human and *Photobacterium leiognathi *SODs with experimental rate constants of 2.5 and 8.5 10^9^M^-1^s^-1^, respectively, at pH7 and 20 mM ionic strength [55]. Open circles : bovine SOD at pH 7.7 under ionic strength values of 20, 40, 90, 160 and 250 mM [26]. Connected filled circles: bovine SOD at 20 mM ionic strength and pH values ranging from 7.7 to 12.3 [26]. All points can be approximated by a linear relation y = 0.45*x (R-coefficient 0.97). The ionic strength dependence alone can be fit with y = 0.3*x (R-coefficient 0.99). On panel (b), experimental rates [26] are shown as filled circles. The pH dependence of the rates is calculated by assigning 2 different values of the dielectric constant of the protein interior (ε), 4 and 78, when computing residue pK_*a *_values (see Methods section). The value of 78 was used for pK_*a *_calculations for all titratable residues and was expected to give better agreement with experiment [28].

For both types of variation of environmental conditions, bovine SOD exhibits a distinct correlation between electrostatic potential differences and differences in the experimental kinetic constant. A 1 ln unit change in kinetic constant corresponds to 0.45 kcal/mol/e change of average electrostatic potential in the region within 10 Å of the catalytic copper ion, see Fig. 4a. This ratio also applies when comparing bovine and human SOD at pH 7 and an ionic strength of 20 mM.

For the bovine SOD above pH 11 and for the *Photobacterium leiognathi *SOD, larger electrostatic potential differences correspond to a 1 ln unit change in kinetic constant. This deviation in pH dependence can be attributed to the difficulty in calculating the pK_*a*_s of amino acid residues of SOD. The pH-dependence of the rate constants of bovine SOD is shown in Fig. 4b. For example, assigning a low dielectric constant (ε = 4) to the protein interior resulted in lower pK_*a *_values of Lys and Arg residues and a too steep drop in the rate constant around pH 9, see Fig. 4b. Significant pH-dependent changes in k_cat_/K_m _are observed experimentally to occur at pH 11. This behavior can be reproduced when electrostatic potentials are calculated using the charges, assigned from pK_*a *_calculations with a protein dielectric constant of 78. Use of this high value of the protein dielectric in computations of the pK*_a_* values of these residues is expected to give the most accurate pK*_a_* values according to the methodology applied [28].

### Molecular electrostatic potential differences correlate with substrate K_m _and k_cat_/K_m _rate constants for a set of triose phosphate isomerases (TPI) from different organisms

The reaction mechanism of TPI has been studied extensively [29] and the consistency of measured kinetic constants has been analyzed [30]. Here, we selected TPIs from 12 different organisms that have K_m _and k_cat _values in the BRENDA database [31]. In the database, some of the kinetic parameters are duplicated and some are inconsistent with each other. Therefore, before applying qPIPSA, the kinetic parameters were analyzed by referring to the original papers, rejecting values measured under very different conditions and favoring cases in which both k_cat _and K_m _originated from the same authors (see Table 2). The results given here are for TPI protein structure models built using SwissModel and Modeller [32] with the "Turbo" modeling protocol (see Methods section). For the k_cat_/K_m _values of TPIs, the comparison region is centered on the oxygen atom of residue L230 in the active site of TPI. A systematic scanning of different regions (considering each accessible atom as a comparison region center) showed that the differences in electrostatic potential in this region most accurately described changes in the kinetic parameter k_cat_/K_m _(see below).

**Table 2:** Kinetic constants for triose phosphate isomerases (TPI) from 12 different organisms with glyceraldehyde-3-phosphate as substrate

Organism	K_m _(mM)*	k_cat _(10^5 ^min^-1^)	k_cat_/K_m _(10^7 ^M^-1^s^-1^)	SwissProt ID Sequence identity to 1r2rA	Conditions, Reference
*Trypanosoma brucei*	0.46 (0.25–0.46)	3.1 (2.6–3.7)	1.120	TPIS_TRYBB 53%	20°C, 100 mM, pH 7.4 [56]
*Trypanosoma cruzi*	0.45	2.7	1.000	TPIS_TRYCR 53%	20°C, 100 mM, pH 7.4 [57]
*Oryctolagus cuniculus *muscle	0.43 (0.32–0.43)	1.86 (1.9–5.1)	0.721	TPIS_RABIT 100%	30°C, 50 mM, pH 7.6 [56]
*Gallus gallus *muscle	0.47	2.56	0.915	TPIS_CHICK 89%	30°C, 100 mM, pH 7.4 [58]
*Saccharomyces cerevisiae*	1.22 (0.62–1.5)	7.9 (1.4–10))	1.080	TPIS_YEAST 52%	25°C, 100 mM, pH 7.6 [59]
*Leishmania mexicana*	0.41 (0.30–0.41)	2.52 (4.3–2.5)	1.020	TPIS_LEIME 52%	25°C, 100 mM, pH 7.6 [60]
*Plasmodium falciparum*	0.35	2.68	1.280	TPIS_PLAFA 42%	30°C, 50 mM, pH 7.9 [56]
*Vibrio marinus*	1.9	4.2	0.368 (0.515)^#^	TPIS_VIBMA 41%	10°C, 100 mM, pH 7.6 [61]
*Escherichia coli*	1.03	5.4	0.874	TPIS_ECOLI 45%	25°C, 100 mM, pH 7.6 [61]
*Homo Sapiens*	0.49	2.7	0.918	TPIS_HUMAN 98%	25°C, 100 mM, pH 7.6 [62]
*Giardia lamblia*	0.53	2.9	0.912	TPI1_GIALA 45%	25°C, 100 mM, pH 7.4 [63]
*Spinacia oleracea*	0.68	2.7	0.662	TPIC_SPIOL 60%	25°C, 100 mM, pH 7.5 [64]

* The range of values extracted from BRENDA is given in brackets.

^# ^The value after correction to 25°C obtained by multiplying by 1.4 (the ratio of water viscosity at 10°C compared to 25°C).

**Figure 5:**
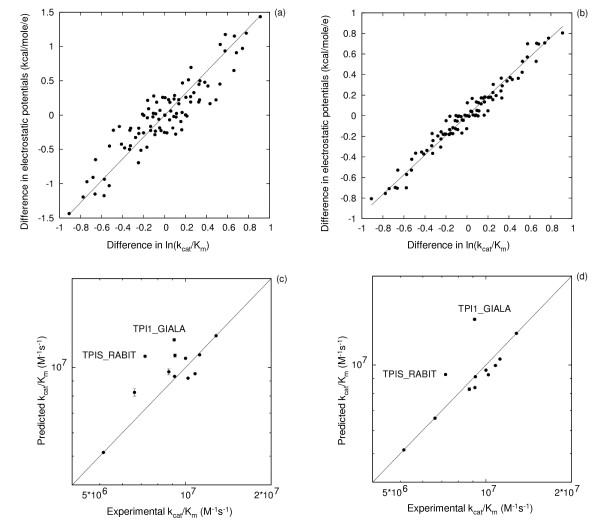
**(a and b) Correlation between experimental ln(k_cat_/K_m_) and electrostatic potential differences for TPI and (c and d) prediction of k_cat_/K_m _for TPI for the substrate glyceraldehyde-3-phosphate**. Correlations (a and b) are for differences in ln(k_cat_/K_m_) and electrostatic potential differences of each TPI protein pair, after excluding TPI1_GIALA and TPIS_RABIT. The straight lines correspond to the best linear fit and are given by y = 1.59*x (a) and y = 0.95*x (b). Predictions (c and d) were made for 10 TPIs based on experimental measurements for the two TPIs (from *V. marinus *(TPIS_VIBMA) and *P. falciparum *(TPIS_PLAFA), see Table 2) at the minimum and maximum points on the y = x line, respectively. These 2 cases were selected to derive the correlation factor α in equation (1). Then this factor was used to compute the kinetic parameter in the other cases – there are 2 predictions for each case and their deviation defines the error bars. Two outliers (with errors greater than 1.5 RMS deviation from y = x) are labeled, see text. Correlations and predictions for (a) and (c) were done using Modeller protein structural models, whereas SwissModel models were used for the correlations and predictions presented in (b) and (d).

For the k_cat_/K_m _parameter, we find that an increase of k_cat_/K_m _of 1 ln unit is related to a ca. 1.59 kcal/mol/e increase of average electrostatic potential, see Fig. 5a,b. The opposite dependence compared to AChE reflects the fact that for TPI the substrate has a negative -2e charge, whereas for AChE, TFK+ and ATCh have a +1e charge.

This correlation can be used to predict enzyme kinetic parameters of triose phosphate isomerases. The kinetic parameters from species with the highest and lowest k_cat_/K_m _values were used to calculate and predict parameters for the remaining 10 species, see Fig. 5c and 5d. In general, a satisfying agreement between calculated and experimental values was obtained. The relative ordering of all species could be reproduced. Computation of the k_cat_/K_m _parameters for the remaining 10 TPIs shows that the TPIs (from rabbit and *Giardia lamblia*) appear to be outliers, see Fig. 5c and 5d. The predicted k_cat_/K_m _values for enzymes from these organisms were significantly larger than those measured experimentally. The correlations were better for the SwissModel models (Fig. 5b and 5d) than for the Modeller "Turbo" models (Fig. 5a and 5c).

**Figure 6:**
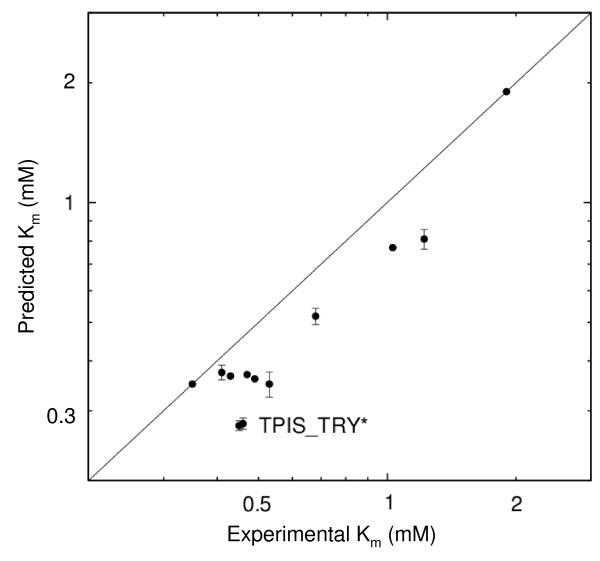
**Prediction of K_m _values for TPI for the substrate glyceraldehyde-3-phosphate**. Predictions were made for 10 TPIs based on experimental values for 2 TPIs (from *V. marinus *(TPIS_VIBMA) and *P. falciparum *(TPIS_PLAFA). Two outliers (with errors greater than 1.5 RMS deviation from y = x) are labeled by TPIS_TRY*, referring to the enzymes TPIS_TRYBB and TPIS_TRYCR, see Table 2).

In a similar manner, enzymatic K_m _values can be predicted from a correlation between electrostatic potential differences and experimental K_m _values. In this case, the region around residue W168 was chosen to calculate potential differences as changes in the potential were found to describe changes in the K_m _value most accurately in this region (see below). A 0.85 kcal/mol/e increase in electrostatic potential results in a 1 ln unit decrease in K_m _value. (In the region around L230O, a 0.94 kcal/mol/e increase in potential results in a 1 ln unit decrease in K_m _value). Based on the two extrema, the K_m _values of the remaining species can be computed (Fig. 6). The relative ordering of K_m _values of all species could be reproduced.

In TPIs, the substrate has a net charge of opposite sign and twice the magnitude of the substrate of AchE. Therefore the correlation coefficient should be expected to have approximately half the magnitude and an inverted sign compared to the AchE case, in accord with its physical meaning, described in the Theory section. This is indeed the case for the correlation between the electrostatic potential differences and K_m _values.

### Molecular electrostatic potential correlates with substrate K_m _and k_cat_/K_m _values for Class I Fructose 1,6-bisphosphate aldolase (FBA) when an appropriate conformation of the C-terminal part of the protein is used

Fructose-1,6-(bis)phosphate aldolase is a ubiquitous enzyme that catalyzes the reversible aldol cleavage of fructose-1,6-(bis)phosphate to glyceraldehyde-3-phosphate and glycerone phosphate. Class I aldolases of animals and higher plants use covalent catalysis through a Schiff-base intermediate. Class II aldolases of most bacteria and fungi require a divalent metal cation as a cofactor [33].

**Figure 7:**
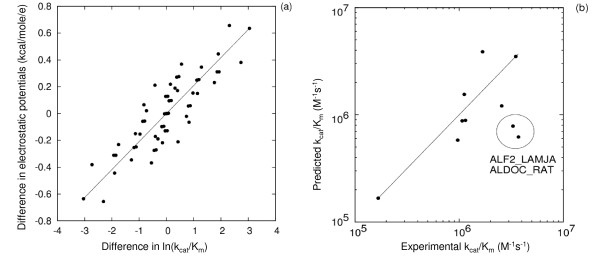
**Correlation between experimental ln(k_cat_/K_m_) for bisphosphate and electrostatic potential differences for different aldolases (a) and prediction of k_cat_/K_m _for bisphosphate and class I aldolase (b)**. Correlations (a) are for differences in ln(k_cat_/K_m_) and electrostatic potential differences of each aldolase protein pair, after excluding ALF2_LAMJA and ALDOC_RAT. Predictions presented on part (b) were made for 8 aldolases based on ALDOB_RABIT and Q9U5N6_LEIME experimental parameters. Two outliers (with errors greater than 1.5 RMS deviation from y = x) are labeled, see text for discussion.

Kinetic data for 10 different class I fructose 1,6-bisphosphate aldolase enzymes and isozymes from 6 different species are available from the BRENDA database [31], see Table 3. FBA was selected for study here as it is rather well characterized experimentally, with both k_cat _and K_m _data available for the 10 different aldolases. The comparison region was chosen to be a collection of points on the skin of the protein (see Methods section) within 10 Å of the active site of human aldolase A. In order to achieve an unbiased assignment of the active site, the center of this region was determined to be the most probable location of the active site pocket using the Putative Active Sites with Spheres (PASS) tool [34]. Other choices, such as the region around K146NZ or K229NZ, atoms of residues known to be involved in substrate binding and reaction, give similar results. The values of k_cat_/K_m _are highly correlated with electrostatic potentials in the active site. The correlation corresponds to a change of k_cat_/K_m _by 1 ln unit when the electrostatic potential changes by 0.21 kcal/mol/e, see Fig. 7a. This value is smaller than the 1.59 kcal/mol/e for TPI. This can be explained by considering that the substrate in this case is bisphosphate with 2 phosphate groups and a -4e charge at pH 7.

**Table 3:** Kinetic data for 10 different Class I Fructose-1,6-bisphosphate aldolases (FBA) with fructose-1,6-bisphosphate as substrate

Organism	K_m _(mM)	k_cat _(s^-1^)	k_cat_/K_m _(10^6 ^M^-1^s^-1^)	SwissProt ID Sequence identity to 1zaiA	Reference
*Trypanosoma brucei*	0.0092	10.3	1.12	ALF_TRYBB 49%	[65]
*Oryctolagus cuniculus*	0.0095	10.2	1.07	ALDOA_RABIT 100%	[35]
*Oryctolagus cuniculus*	0.00084	2.94	3.50	ALDOB_RABIT 71%	[35]
*Entosphenus japonicus*	0.018	46.1	2.56	ALF1_LAMJA 75%	[66]
*Entosphenus japonicus*	0.0048	15.8	3.29	ALF2_LAMJA 74%	[66]
*Leishmania mexicana*	0.049	8.2	0.167	Q9U5N6_LEIME 49%	[67]
*Rattus norvegicus*	0.0089	33	3.71	ALDOC_RAT 81%	[68]
*Homo sapiens*	0.052	59.7	1.15	ALDOA_HUMAN 98%	[68]
*Homo sapiens*	0.012	20.1	1.68	ALDOB_HUMAN 69%	[68]
*Homo sapiens*	0.013	12.6	0.969	ALDOC_HUMAN 82%	[68]

**Figure 8:**
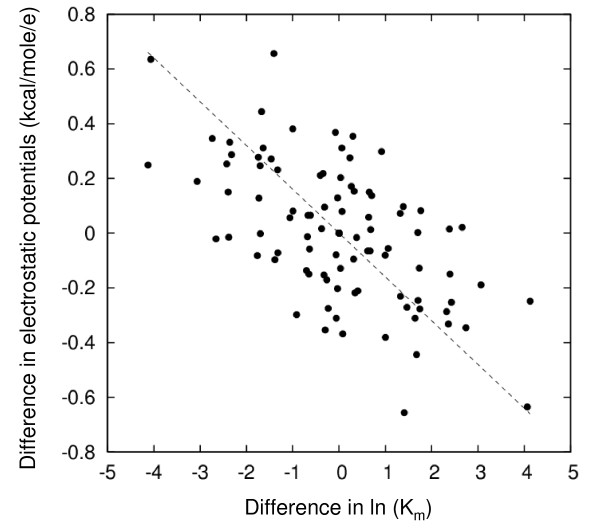
**Correlation between experimental ln(K_m_) for bisphosphate and class I aldolase electrostatic potential differences**. Correlations are for differences in ln(K_m_) and electrostatic potential differences for each aldolase protein pair. All possible pairs of the 10 aldolases are compared.

When making predictions for 8 of the aldolases based on known k_cat_/K_m _values for two aldolases, it is found that two proteins, ALF2_LAMJA and ALDOC_RAT, appear as outliers, see Fig. 7b. In both cases, the k_cat_/K_m _values are predicted to be more than 4 times lower than the experimental values. This deviation is outside the uncertainty of the qPIPSA method and points to either the need for additional experimental assays or to additional contributions to the catalysis of these two enzymes. Removing the outliers changes the R-coefficient of correlations between experimental and predicted ln(k_cat_/K_m_) from 0.63 to 0.87. The K_m _values for aldolases also show a detectable correlation with electrostatic potentials – 60% error defined by formula (3) and R-coefficient -0.59, see Fig. 8.

Aldolases present a rather complicated case for the analysis based on the electrostatic potential in the active site region because it is known that the kinetic parameters of aldolases are modulated by a flexible C-terminal part as well as isozyme specific residues that are not located in the active site of the enzyme [35,36]. Nevertheless, kinetic parameters correlate with the interaction properties near the active site of the enzyme. The electrostatic potential changes here have a much larger impact on K_m _than in the other cases investigated in this manuscript: changes of electrostatic potential by 0.3 kcal/mol/e change K_m _by 1 ln unit. As was already mentioned, this can be attributed to a larger charge of the substrate in this case. At the same time, there is a possibility that putative conformational rearrangements, that are not included in our modeling, are accounted for by electrostatic potential changes implicitly – conformational changes could cause further changes in electrostatic potential and these changes could account for changes in K_m _directly by contributing to the interaction with the substrate. We also find that, in this case, the selection of a reference structure with an appropriate conformation is very important. The correlation was lost when we used the structure of the human aldolase A (muscle-type) with PDB code 1ALD instead of the structure of the rabbit muscle aldolase A (PDB code 1ZAI) as a template, even though these proteins have 98% sequence identity. The main difference between the two crystal structures is in the conformation of C-terminal residues: in 1ALD, the C-terminal forms part of the active site of the enzyme [37] whereas in 1ZAI, it does not. The critical involvement of the C-terminal loop in catalysis has been noted before. C-terminal truncated aldolases are practically catalytically inactive. See also reference [38].

### A conservative comparative modeling protocol is required for correlating MIFs and kinetic parameters

**Figure 9:**
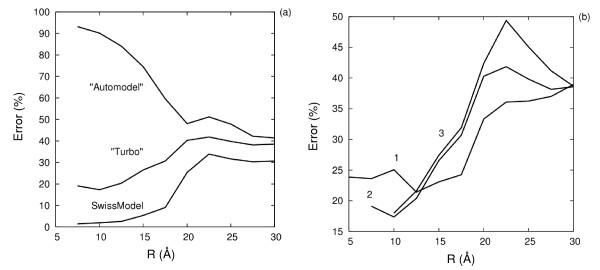
**Correlation errors for ln(k_cat_/K_m_) for TPI for (a) different protein structural models and (b) different specifications of the MIF comparison region**. (a) The relative error of predicted ln(k_cat_/K_m_) values of protein structural models generated by 3 different modeling protocols (see Methods for details) as a function of radius R of the sphere of comparison (with error defined as in expression (3) of the Theory section). Calculations were done with a skin accessible to a probe of radius δ = 2 Å. (b) Relative error in correlation of protein structural models using the ''Turbo'' protocol in Modeller with different probe radii δ in Å (see Methods). The radius of the comparison region, R, is varied from 7.5 to 30 Å and it is centered on the atom L230O. The degree of correlation is described by the relative correlation error, formula (3).

The results described above for TPI are for protein structure models built using Modeller [32] with the "Turbo" modeling protocol (see Methods section). Other modeling protocols were tried and it was found that the modeling accuracy is important for establishing good correlations. We compared two protocols: the "Automodel" mode of Modeller with extensive optimization of the structures and the "Turbo" mode, in which only molecular mechanics minimization of the structures after building the initial homology model (based on a single template) is performed. There is a ca. 1 Å RMS deviation of Cα atoms between these models, but the side chain orientations are more variable in the "Automodel" models. The models showed different degrees of correlation of the electrostatic potential difference and ln(k_cat_/K_m_) values, when the region radius, R, was varied from 7.5 to 30 Å. The region itself is a collection of points on a "skin" of 3 Å thickness and accessible to a probe of 1, 2 or 3 Å radius within a distance R from the center, here atom L230O, see Fig. 9a.

When a probe radius, δ, of 1 Å was used to define the skin, the correlation error was roughly constant up to R = 18 Å and then abruptly increased for larger values of R. The use of a probe radius of 3 Å gave errors systematically increasing with R for "Turbo" models, and decreasing and reaching a minimum for "Automodel" models. The smallest correlation error, 17%, was obtained at a probe radius of 2 Å and "skin" thickness of 3 Å with "Turbo" models at R = 10 Å. "Automodel" models only gave a correlation error minimum of ca. 50% and this was obtained at R = 20 Å. This shows that the orientation of side chains is important for obtaining correlations. Inconsistent side-chain positioning, as in the "Automodel" case, reduces the possibilities for deriving a correlation between the structure and the kinetic parameters. Nevertheless, a weak correlation can still be identified by using a larger radius R for the comparison region so that errors due to side chain conformational differences are partly cancelled out.

**Figure 10:**
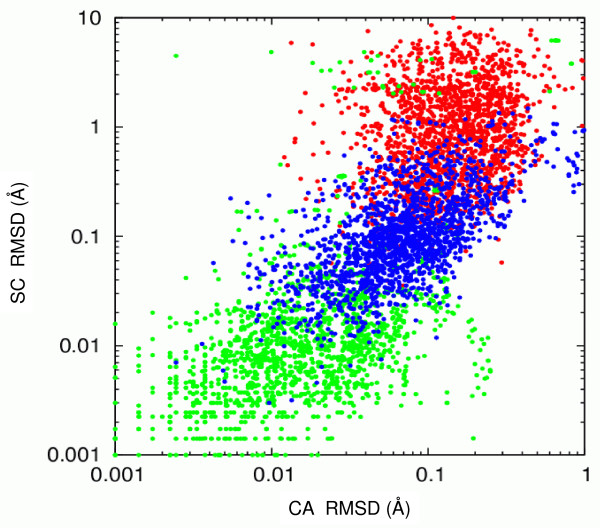
**Comparison of three different protein structure modeling protocols for modeling TPIs**. The RMSD of atoms of charged side chains is plotted against the RMSD of Cα atoms of equivalent residues in all pairs of TPI models. The data for three different sets of models are presented in green (SwissModel), blue (Modeller "Turbo") and red (Modeller "Automodel"), see Methods for details. The charged side chain atoms selected for RMSD calculations are Cζ, Nζ, Cγ and Cδ of the residues ARG, LYS, ASP and GLU, respectively. This plot shows clear differences between the three sets of models: the charged side chain atom RMSDs are on average 0.01, 0.1 and 1.0 Å for the SwissModel, ''Turbo'' and ''Automodel'' Modeller models, respectively, even though the Cα RMSDs differ insignificantly, being less than 1 Å in all three sets.

We also tested the models obtained by modeling with SwissModel [39]. We were able to model all 12 TPIs using the the SwissModel web-server, although the web-server does not produce any model when the sequence identity is too low or modeling problems are encountered. The SwissModel models differ from the Modeller "Turbo" models by the same amount (in terms of RMSD of Cα atoms) as the Modeller "Automodel" models, but the SwissModel models have more ordered side chain conformations than the Modeller "Turbo" models, see Fig. 10. The correlations derived from the SwissModel models were better than the correlations found for the Modeller "Turbo" models, although differences in RMSDs were very small (Fig. 10).

### Correlation of kinetic parameters depends on the comparison region and this dependence may give insights into determinants of kinetic parameters

In comparing MIFs and correlating their differences with kinetic parameter differences, a comparison region must be chosen. An appropriate size, shape and location are not obvious, although one can expect that the comparison region should be near the active site of the enzyme. We already noticed that the comparison region should neither be too small nor too large (see Materials and Methods section for a detailed description), a region with a radius of approximately 10 Å is optimal (see Fig. 9).

In the case of AChE, comparisons over a single region in the gorge near the buried active site result in correlations for the inhibitor association rate constant as well as the substrate k_cat_/K_m _and K_m _parameters. Moving the comparison region to the active site results in slightly poorer correlations. Moving the comparison region to the entrance of the gorge results in significantly poorer correlations for the majority of cases. This means that the electrostatic potential closer to the active site is of more importance for the kinetic parameter values, i.e. rate-determining. At the same time, the contribution from the surface residues to the potential in this region does not fully account for the contribution of these residues to the inhibitor association rate constant (Fig. 2a). This means that the interaction field in this region does not fully describe the kinetics. However, in this case a single region still describes kinetic parameters quite accurately.

**Figure 11:**
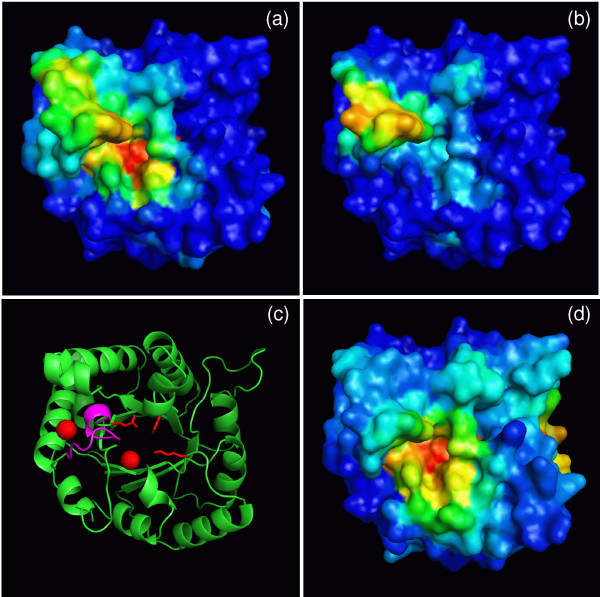
**Identification of regions on the surface of TPI relevant for k_cat_/K_m_ and K_m_ kinetic parameters**. By scanning patches and residues on the protein surface, calculating the correlation between electrostatic potential differences and the respective kinetic parameters k_cat_/K_m _(a) and K_m _(b) at each position, different regions on the TPI protein surface were found to give the best correlations with experimental data. Regions where variations in the electrostatic potential are best correlated with variations in k_cat_/K_m _(a) and K_m _(b) (see text for details). (c) ribbon presentation of the TPI monomer with important residues shown: the flexible loop is in magenta (W168-T175), the catalytic residues Glu165 and His95, as well as the conserved Lys13 important for electrostatic steering of the substrate are shown in red. The centers of the regions selected for correlating K_m _and k_cat_/K_m _parameters are shown by red balls, on the left – W168CZ3 and on the right – L230O. (d): electrostatic potential conservation: red: highest to blue: lowest.

In the case of TPI, the best correlations are obtained for different comparison regions for the two different kinetic parameters, K_m _and k_cat_/K_m_. The region best describing k_cat_/K_m _is located near the active site (Fig. 11), while variations in K_m _value are better described by the region around the flexible loop of TPI that can close over the active site for the reaction. The hinge residues have been shown by mutagenesis to be important for the reaction catalyzed by TPI [40,41].

It can be expected that the same region is responsible for parameters correlated with each other. The appearance of two different regions responsible for 2 different kinetic parameters is expected when the 2 different kinetic parameters are not correlated with each other, as in case of TPI, because the interaction field changes in a single region can describe only one set of parameters. The region responsible for the TPI parameter k_cat_/K_m _can be interpreted as contributing to the interaction between the substrate and the enzyme, because it is located near the substrate binding position. The region responsible for K_m _is close to the active site, but one cannot expect that the substrate interacts with the enzyme there, because the region is on the other side of the flexible loop from the active site. One can assume that the interaction field variations define K_m _variations indirectly. It is the variation of physico-chemical properties of the residues at the beginning of the flexible loop that changes K_m _values, but we see these variations as variations of the electrostatic potential around W168.

## Conclusion

We have shown that protein interaction field differences can correlate with changes in the kinetic parameters of enzymes. The application possibilities of this observation are two-fold. First, the correlation method can be used to check the consistency of kinetic measurements with available structural data. In the case that the predictions are significantly different from measured parameters, significant structural changes can be expected or the measurement conditions are not consistent with the structure of the enzyme. Second, prediction of kinetic parameters is possible when data consistency is present – given two enzymes with known kinetic parameters and structure, one can derive a correlation and make predictions for the rate constants of a similar enzyme with known structure. Two is the minimum number of known parameters required for application of the method. For error estimates, more known kinetic parameters are needed and the more measured kinetic parameters are used, the more reliable the predictions will be.

An important area of application is the use of protein three-dimensional models to bring experimental measurements into consistency with each other. Measurements of kinetic constants are frequently done under different conditions and often result in different values for the same parameter. Here, the structural properties can be used as a reference point for rationalising two different measurements.

The generality of the qPIPSA method is that it uses a concept of similarity that does not require *a priori *knowledge of the specific mechanism of the enzymatic reaction. Although only consideration of the detailed mechanism can permit quantification of all the contributions, general structural differences can explain the differences between the enzymes resulting in their different kinetic properties. This is analogous to the case of protein association, in which relative association rates of protein mutants are described well by differences in interaction energies calculated in a simple way [42], although the association process itself may be changed significantly by mutations [43].

The consistency of kinetic measurement conditions and structural data is dependent on the accuracy of structural modeling. The requirement for protein structural model accuracy is in general high. It varies according to the type of parameter. k_on _or k_cat_/K_m _values, for which long-range interactions are important may be less sensitive to structural details than k_cat _which is defined by short-range interactions. In our study, we avoided the problem of rotating side-chains by forcing equivalent side-chains to have the same conformation. This approach may overlook parameter changes due to side-chain conformational changes, but as shown here, in many cases it allows significant correlations between structural and kinetic parameters to be detected. Improvements in homology modeling methods [44] may result in improvements in this type of prediction.

The minimum requirements for performing qPIPSA are (i) that a structural model of the enzyme of adequate quality for computation of the electrostatic potential in the region of the active site must be available or modellable, and (ii) that enzymatic kinetic parameters for related enzymes having the same reaction mechanism must be available. According to our estimates, a significant fraction of characterized enzymes satisfy these requirements and as such can be investigated by qPIPSA. Factors influencing the validity of qPIPSA include the following. (i) The relevance of the protein structures analysed for the rate-determining catalytic step. Proteins may adopt multiple conformations. Sometimes it may be necessary to do calculations with more than one conformation, e.g. with open and closed forms of an enzyme active site. If one of these gives MIFs that correlate better with known kinetic parameters, this provides some mechanistic information about the determinants of the kinetic parameter. (ii) Similarly, the region over which to compare the MIFs should be chosen. Calculations can be done for a number of regions. For TPIs, the best correlation with kinetic parameters was obtained for different regions for k_cat_/K_m _values and K_m _values. This again provides some mechanistic information on the parameter determinants. (iii) The method is most suitable when the rate-determining step is mechanistically the same across the set of protein structures compared. An outlier might be mechanistically different, but if there is wide mechanistic variation in the dataset, the comparative approach cannot be expected to work. (iv) Molecular dynamics are not currently considered in the approach and if they alter the protein structures in different ways across the dataset they will adversely affect the value of the MIF comparison.

In summary, we have described qPIPSA and demonstrated its validity for establishing correlations between kinetic parameters and MIFs, as well as estimating kinetic parameters using MIFs computed from enzyme structures. qPIPSA can be a useful tool for parameter estimation in biochemical network modelling in systems biology applications as well as in investigating enzymatic structure-function relationships and enzyme mechanisms.

## Methods

### Modeling of protein structures

All protein structures were modeled by comparative modelling techniques with SwissModel [39]. In addition, Modeller [32] was used to model TPI structures. Comparative modeling techniques are suitable as the level of sequence identity between the template protein with experimentally determined structure and the modeled protein was rather high, being greater than 40% for all cases studied.

One X-ray crystal structure was chosen as the template for each enzyme family (with PDB and subunit identifiers as follows: 1mahA for AChE, 1cbjA for SOD, 1r2rA for TPI, 1zaiA for FBA). TPI and SOD are homodimers, but monomeric models were used here, as there is little interweaving of the monomer structures. Only one monomer of FBA was used for the same reason, although the majority of aldolases are homotetramers.

For a given enzyme family, all comparative models were built from one single template crystal structure even when other crystal structures were available with higher sequence similarity to the modeled protein. This was done to ensure that the differences between the models of different proteins of one enzyme family were due to differences in sequence only and not influenced by differences in crystallization conditions of different template crystal structures. Such differences can, for example, result in different rotamers of flexible side chains being seen in different structures corresponding to the same protein sequence.

For Modeller, as well as using the default mode "Automodel", we used a modeling procedure called "Turbo", to avoid excessive optimization of models. Excessive optimization and MD refinement can result in differences between models of different proteins in one enzyme family that do not reflect the sequence differences.

The "Automodel" class in Modeller8v.1 [32] provides a convenient set up of default parameters for the generation of homology models. By default, protein structural models are energy minimized and then subject to a molecular dynamics and simulated annealing procedure to overcome bad contacts or local minima. A detailed description of the procedure and settings can be found in the Modeller documentation . The CHARMM22 [45] force field for proteins is used with modified dihedral parameters. Energy minimization is done using a conjugate gradients optimisation method for a given maximum number of iterations. The minimal atomic shift for the optimisation convergence test is 0.010 Å. The molecular dynamics integrator uses the Verlet algorithm; the time step in MD simulations is set to 4 fs.

The default (referred to here as "Automodel" procedure) settings correspond to a minimization for a maximum of 200 steps, followed by a "very_fast" MD and simulated annealing simulation. Initial models are randomised in their xyz coordinates by adding a random number between -4.0 and +4.0 Å to the initial coordinates. MD equilibration is performed at 150, 400 and 1000 K for 50 iterations each. Simulated annealing is done at 1000, 800, 500 and 300 K for 300 iterations at each temperature.

The simplified protocol (referred to here as the "Turbo" procedure) uses the "automodel.very_fast" pre-defined settings. They differ from the default in that initial coordinates are not randomised; energy minimization is done for a maximum of 50 steps with no subsequent MD and simulated annealing runs. In both cases, the quality of the generated models was checked with WHATCHECK [46] to remove bad models.

We applied different additional protein structure quality verification tools (PROSA [47], Verify3D [48] and ERRAT [49]). These gave little preference to any one of the three modelling procedures.

### Analysis of molecular interaction fields

The PIPSA (Protein Interaction Property Similarity Analysis) [13] software (version 2  with minor modifications) was used to quantify MIF differences. The MIF used was the molecular electrostatic potential. Other MIFs for the interaction of different chemical fragments or functional groups with macromolecules (these can, for example, be computed with the GRID program [50] for a range of probes such as carboxylate, amino and hydrophobic probes), were not used in this work.

PIPSA permits the comparison of the interaction fields for a superimposed protein pair in a region defined by the intersection of the "skins" of these two proteins. The "skin" is defined as a region outside the protein surface, accessible to a probe of radius δ, and inside the surface accessible to a probe of radius δ + σ; the "skin" thus has a thickness σ. The comparison region can be restricted to be within a specified distance from a given point, for example, a chosen atomic center. Using PIPSA, we have previously carried out large scale classification of Plekstrin Homology (PH) domains [12], WW domains [8] and E2 ubiquitin conjugating domains [14] by electrostatic similarity to infer binding characteristics. We have also correlated electron transfer rates with the similarity of electrostatic potentials for a set of plastocyanin mutants [20]. In these studies, a probe of radius ψ = 3 Å and a "skin" of thickness σ = 4 Å were used. In the present study, a probe of radius δ = 2 Å and a "skin" of thickness σ = 3 Å were used. These values are smaller than those used previously because the kinetic parameters of enzymes can be influenced by the interaction fields in the active site close to the enzyme surface. The use of a skin closer to the surface meant that it was important to derive models with consistent side chain orientations (see above).

For each protein structure, the molecular electrostatic potential was computed as follows. Polar hydrogen atoms were added using Whatif [46] and electrostatic potentials were calculated by numerically solving the linearized Poisson-Boltzmann equation using the UHBD program [51] with atomic radii and partial atomic charges assigned from the OPLS parameter set [52]. The grid spacing was set to 1 Å and grid was dimensioned to 110^3 ^points. The dielectric surface of the protein was defined as the molecular surface accessible to a spherical water probe of radius 1.4 Å. The relative dielectric constant of the protein interior was set to 2, the dielectric constant of the solvent was 80. The ionic strength of the solvent was 0 mM in the case of AChE, 100 mM for TPI, 150 mM for FBA and variable for SOD in accord with the corresponding experimental conditions.

In all cases, we used the following protocol to select the MIF comparison region:

1. The skin is assigned a thickness σ of 3Å extending from the probe accessible surface around the protein computed with a probe of radius (δ) 2 Å.

2. The region is assigned a radius of 10Å. If there are fewer than 50 points for comparison in this region, the radius is incremented in 2 Å intervals until enough points are included for reliable calculations.

3. The center of the region is assigned in the active or binding site. How this is chosen depends on the information available on the enzyme under study. If a structure is known of an enzyme-ligand complex, the region can be centered on the ligand. If the catalytic residues are known, the center can be one of these or their geometric center. The comparison region should cover the region in which the ligand interacts with the enzyme, but may also be chosen to include a region relevant for ligand access such as the gorge in AChE.

For AchE, the MIF comparison region was selected to be within 10Å of a point near His447 in the substrate active site gorge [25] (namely, the mid-point between the atomic coordinates of H447ND1 and Y124OH in the X-ray crystal structure with PDB identifier 1mah, subunit A). The spherical region thereby included the active site, the gorge and the peripheral substrate binding site at the gorge entrance. There were about 50 grid points in the 3 Å thick skin extending from the surface accessible to a 2 Å radius probe. Residues Glu202 was protonated and His447 was doubly protonated, as described previously [53].

SOD structures were modeled based on the structure of *Bovine *superoxide dismutase [54]. The pH dependence of protein charges was modeled by calculating the pK*_a_* values of ionisable sites using a Poisson-Boltzmann electrostatics method [28]. Calculations were done for assignments of high (78) and low (4) values of the dielectric constant of the protein. The charges of the Cu and Zn ions and their ligands were assigned as described previously [27] and these charges were not changed with pH. The electrostatic potentials were compared in the "skin" region within 10 Å of the catalytic copper ion. The comparison region in TPI and FBA cases has the same radius with the centers assigned as described in the text.

## Abbreviations

AChE – Acetylcholinesterase

ATCh – Acetylthiocholine

FBA – Class I fructose-1,6-bisphosphate aldolase

K_m _– Michaelis-Menten constant

k_cat _– Turnover number

ln – Natural logarithm

LTO – Leave-two-out cross-validation

MIF – Molecular interaction field

PIPSA – Protein Interaction Property Similarity Analysis

PLS – Partial Least Squares

RMSD – Root mean square deviation

QSAR – Quantitative structure activity relationship

SOD – Superoxide dismutase

TFK+ – *m*-trimethylammoniotrifluoroacetophenone

TPI – Triose phosphate isomerase

## Authors' contributions

RRG carried out the calculations and modeling; MS built the Modeller structural models and assessed the accuracy of modeled structures; RRG, MS and RCW participated in the design and coordination of the study, the analysis of results and the writing of the manuscript. All the authors have read and approved the final version of the manuscript.
